# Combining Environmental Variables and Machine Learning Methods to Determine the Most Significant Factors Influencing Honey Production

**DOI:** 10.3390/insects16030278

**Published:** 2025-03-06

**Authors:** Johanna Ramirez-Diaz, Arianna Manunza, Tiago Almeida de Oliveira, Tania Bobbo, Francesco Nutini, Mirco Boschetti, Maria Grazia De Iorio, Giulio Pagnacco, Michele Polli, Alessandra Stella, Giulietta Minozzi

**Affiliations:** 1 Institute of Agricultural Biology and Biotechnology, Italian National Research Council (CNR), 20133 Milan, Italy; johanna.ramirezdiaz@ibba.cnr.it (J.R.-D.); arianna.manunza@ibba.cnr.it (A.M.); tania.bobbo@cnr.it (T.B.); giulio.pagnacco@unimi.it (G.P.); alessandra.stella@ibba.cnr.it (A.S.); 2 Statistics Department, Paraíba State University, Campina Grande 58429-500, Brazil; tadolive@servidor.uepb.edu.br; 3 Institute for Electromagnetic Sensing of the Environment, Italian National Research Council (CNR), 20133 Milan, Italy; nutini.f@irea.cnr.it (F.N.); boschetti.m@irea.cnr.it (M.B.); 4 Department of Veterinary Medicine and Animal Sciences (DIVAS), University of Milan, 26900 Lodi, Italy; michele.polli@unimi.it (M.P.); giulietta.minozzi@unimi.it (G.M.)

**Keywords:** *Apis mellifera*, honey production, machine learning, prediction, environmental conditions

## Abstract

Bees are crucial for food production and biodiversity. However, extreme weather variation and harsh winters are the leading causes of colony losses and low honey yields. This study aimed to identify the most important features and predict Total Honey Harvest (THH) by combining machine learning (ML) methods with climatic conditions and environmental factors recorded from the winter before and during the harvest season. The initial dataset included 598 THH records collected from five apiaries in Lombardy (Italy) during spring and summer from 2015 to 2019. Colonies were classified into medium-low or high production using the 75th percentile as a threshold. A total of 38 features related to temperature, humidity, precipitation, pressure, wind, and enhanced vegetation index–EVI were used. Three ML models were trained: Decision Tree, Random Forest, and Extreme Gradient Boosting (XGBoost). Model performance was evaluated using accuracy, sensitivity, specificity, precision, and area under the ROC curve (AUC). All models reached a prediction accuracy greater than 0.75 both in the training and in the testing sets. Results indicate that winter climatic conditions are important predictors of THH. Understanding the impact of climate can help beekeepers in developing strategies to prevent colony decline and low production.

## Simple Summary

Honey bees are essential for food production and biodiversity, but extreme weather events and harsh winters can significantly affect bee colonies and reduce honey yields. This study investigated if winter weather could be used to predict honey production in the subsequent harvest season. By analyzing environmental data from both winter and harvest seasons, using variables such as temperature, humidity, precipitation, pressure, wind, and vegetation, three different machine learning (ML) models were used to predict honey yields. Data were collected from five Italian apiaries within a breeding population to train the models from 2015 to 2019. Model performance was evaluated using accuracy, sensitivity, specificity, precision, and area under the ROC curve (AUC). The results showed that winter weather conditions have a substantial impact on honey production and can serve as predictors. Understanding and forecasting these patterns can assist beekeepers in making informed decisions to protect their colonies and optimize honey yields.

## 1. Introduction

There is a close relationship between honey production and climate. Honey production is the consequence of a complex social organisation with specific external and internal actions that depend on local environmental and geographical conditions. Honey bee flight activity, waggle runs frequency, honey stores, queen provisioning, egg-laying rate, and development are influenced by environmental factors [1,2,3,4]. In addition, latitude and altitude determine the biology, behaviour and distribution of honey bees and flowers.

Temperature, solar radiation, wind, precipitation, and humidity are the most important factors influencing honey harvest [5,6,7,8]. Among the environmental factors, temperature is a determinant of colony development. Indeed, brood growth is influenced by hive temperature, which must remain between 33 and 36 °C and with high relative humidity. The thermoregulation mechanism is triggered when the temperature is above 25 °C; nevertheless, prolonged high (>40 °C) or low (<6 °C) temperatures can result in severe colony losses [9]. Similarly, foraging activity peaks when the temperature ranges between 12 and 25 °C and ceases when the temperature is below −7 or above +43 °C [9,10]. In tropical countries, precipitations strongly affect honey production; indeed, pollen foraging is highly influenced by summer rainfall and cloud cover [11]. In addition, high rainfall during winter has been associated with decreased food storage and increased colony mortality in the Mediterranean islands [9]. Furthermore, since relative humidity levels within the hive influence egg hatching, precipitation is also thought to influence honey productivity and bee survival. In the Mediterranean islands, wind speed can significantly affect flight activity [9], which is related to the increased time spent foraging [12]. In addition, climatic conditions during winter have been associated with health problems, lower reproductive rate, irregular egg laying, condensation starvation, and mortality of honey bees [13]. Therefore, estimating and minimising the impact of winter climatic conditions on the following harvest season is one of the challenges for beekeepers.

Machine Learning (ML) is a branch of artificial intelligence (AI) that refers to the process of implementation and evaluation of algorithms that allow pattern recognition, classification, and prediction by fitting models derived from existing data [14]. Data learning can occur through three approaches: supervised learning, unsupervised learning, and reinforcement learning [15]. Supervised learning algorithms allow the identification of meaningful relationships between a set of input variables (features or predictors) and the label (target or outcome) to be predicted and exploit this information to train different prediction models and evaluate their predictive performance on unknown unlabelled data. In general, this approach includes a training and a testing step. In the first step, a larger training subset is used as input, in which features are learned by the algorithm utilized to build the model. Once trained, the model can be used to make predictions on new data and assess the error during the testing step [16].

Among the several supervised learning methods that can deal with both classification and regression problems, linear models are the simplest and most commonly used ML models. Most linear models are used to solve regression issues, although linear models for classification problems do exist, e.g., logistic regression [15]. Decision trees (DT), non-parametric methods based on root and leaf nodes, are also widely used. These algorithms provide accurate estimates for both large and small datasets and present several advantages over linear methods as no assumptions about the distribution of explanatory variables are required, and they are not influenced by high correlation among independent variables, outliers, and missing values [17,18]. Unlike DT, Random Forest (RF) does not rely on a singular decision, but on several decisions. The greater number of trees in the RF algorithm leads to higher accuracy and prevents overfitting problems [19]. Novel approaches have recently been included in the family of gradient boosting algorithms, e.g., eXtreme Gradient Boosting–XGBoost, which is widely used due to its computational efficiency (fast and lower memory use), high accuracy, and its combination with DT [20,21]. The idea behind the boost is to sequentially build new models in sequence. In essence, “boosting” increases its performance by continuing to build new trees, where each new tree in the sequence attempts to correct where the previous one made the largest errors.

In complex biological data, different ML methods have been successfully used in ecology [22,23], environmental monitoring [17], plant species identification [24,25], flowering time [26,27], and animal science [18,28,29]. ML models have also been used to predict crop yields using honey bee census data, along with climatic and satellite-derived variables [30]. In honey bees, ML methods have been applied for predicting demographic alterations [31], toxicological assessment [32], beehive monitoring [33], varroa infestation [34], behaviour [31], genetics [35,36], queen body mass [37] bee products quality assessment [38,39,40], bee colony health monitoring [41,42], climate change impact and honey production [43,44,45,46,47]. In particular, predictors used for honey yield forecasting were management factors [37], historical weather variables (e.g., rainfall, temperature, and relative humidity) excluding data concerning the year to forecast [45] or several climatic and satellite-derived vegetation data concerning both preceding (11 months ahead) and during the main honey flow period [48].

In Italy, with the honey harvest season starting in March, giving beekeepers a prediction of a subsequent good or poor harvest season using climatic information from the few preceding months will help them design appropriate management strategies. This is particularly important in a country with widespread migratory beekeeping, where colonies are transported to follow blooming patterns. This practice accounts for 39% of all beekeeping operations and 58% excluding hobbyists [49]. Thus, climatic information of winter prior to harvest combined with enhanced vegetation index -EVI, a satellite-derived measure of vegetation greenness during harvest season, were used to determine the most important features and predict the Total Honey Harvest (THH) using ML methods.

## 2. Materials and Methods

### 2.1. Dataset Construction and Variables Pre-Processing

A total of 598 THH records were collected from spring 2015 to summer 2019 from five commercial apiaries located in Monza and Brianza province of the Lombardy region in Italy. The bees belonged to an Italian breeding population composed of a combination of *Apis ligustica, Apis carnica,* and *Buckfast* subspecies. Over a four-year period, these bees were selected in a controlled mating system, focusing on: honey production, gentleness and hygienic behaviour. Further details on the selection scheme can be found in De Iorio et al. [50]. The dataset also included geographic coordinates of apiaries and year of production. The latitude and longitude values were used to retrieve pre-harvest winter (December–February) monthly climatic data from weather stations using the NASA Prediction of Worldwide Energy Resource (POWER) Data Access Viewer [51]. Thus, a total of 33 variables associated with temperature (12), humidity (6), precipitation (3), pressure (3), and wind (9) relative to the winter months prior to the harvest season of the years from 2014 to 2019 were added and used for further analysis (Table 1).

Neither the data distribution nor the residual distribution was analysed because ML methods do not assume a functional distribution and focus on prediction [18].

The continuous THH outcome was classified into high or medium-low production according to the 75th percentile of the THH distribution, where values above 31.2 kg were considered high (set as 1) and values below or equal to 31.2 as medium-low (set as 0). Records with missing climatic and EVI values and THH lower than 5 kg were removed. After editing, 394 THH records were included in the analysis.

### 2.2. Machine Learning Procedures and Model Evaluation

**Figure 1:**
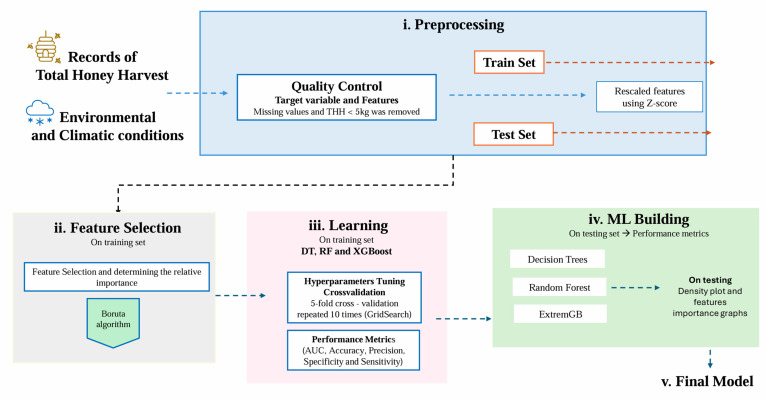
Flowchart illustrating the steps in the full ML pipeline, including pre-processing, feature selection, and model building.

Prediction models were developed using three ML algorithms: DT, RF, and XGBoost. The dataset of 394 records was split into two subsets: 75% of the data (294 records) was used to train the models, while the remaining 25% of the data was excluded from model building and preserved as an external testing set, simulating new unknown data for evaluating model performance. Figure 1 summarizes the ML analysis flowchart used to identify the most important features and predict total honey harvest from climatic and environmental conditions before and during harvest.

Prior to model building and training, features were rescaled using the z-score, and automatic feature selection was performed using the Boruta algorithm in R software [52], which evaluates the relevance of the features and ranks them according to their importance [53,54]. Indeed, Boruta is a feature selection algorithm that iteratively removes the features that are statistically less relevant than random probes and provides an unbiased and stable selection of important and unimportant variables. In particular, Boruta is a wrapper around an RF classifier and uses shifting with fictitious (shadow) characteristics produced throughout numerous iterations to locate all relevant predictor variables. Model training and hyperparameters optimization were computed by applying a 5-fold cross-validation repeated 10 times. In the cross-validation process, the data were divided into five mutually exclusive subsets or folds, and in each iteration of the cross-validation, one fold was reserved for validation, while the remaining four folds were used for model training.

The GridSearch technique was used in order to choose the optimal combination of hyperparameters [55]. The predictive ability of each model on the training and testing sets was then evaluated based on performance metrics like:

(1) $$Accuracy=\frac{True\;positives+True\;negatives}{True\;positives+False\;positives+True\;negatives+False\;negatives}$$

(2) $$Specificity=\frac{True\;negatives}{True\;negatives+false\;positives}$$

(3) $$Sensitivity=\frac{True\;positives}{True\;positives+False\;negatives}$$

(4) $$Precision=\frac{True\;positives}{True\;positives+False\;positives}$$

Besides the metrics above, the area under the Receiver Operating Characteristic (ROC) curve (AUC) was also obtained with the R package yardstick [56]. An AUC of 1 indicates that the classifier can correctly differentiate between all positive and negative class points. An AUC of 0 indicates that the classifier incorrectly classifies all negatives as positives and vice versa. In the present study, we used AUC as the main metric for model selection. In addition, the contribution of each variable to the best model (i.e., feature importance) was computed using the Ranger package [57]. All analyses were performed using the R software version 4.1.3, and ML analysis used the ecosystem package “tidymodels” [58]. For clarity, Supplementary File SA provides pseudocode outlining the key methodological steps.

## 3. Results

### 3.1. Descriptive Statistics

A first exploratory data analysis, including descriptive statistics (mean, standard deviation, and interquartile range), was carried out on the entire dataset (Table 2). The average honey harvest was 23.4 kg, with substantial variability (SD = 11.4 kg). The wide range between the minimum (5.2 kg) and maximum (63.2 kg) harvests highlights the potential influence of environmental factors. The winter months show variations in temperature, humidity, precipitation, and wind speed. Notably, December (T2M_12) experienced average temperatures around 4.5 °C, while January (T2M_1) and February (T2M_2) were slightly cooler and warmer, respectively. Precipitation was highest in February. The EVI, a measure of vegetation greenness, generally increased from March to July, indicating growing vegetation during the honey harvest season. April and June had the highest median EVI values. This suggests a possible link between late-winter precipitation and forage availability during the honey harvest season. Additionally, other factors without available information, such as bee colony health during this period, could also play significant roles.

### 3.2. Features Selection

**Figure 2:**
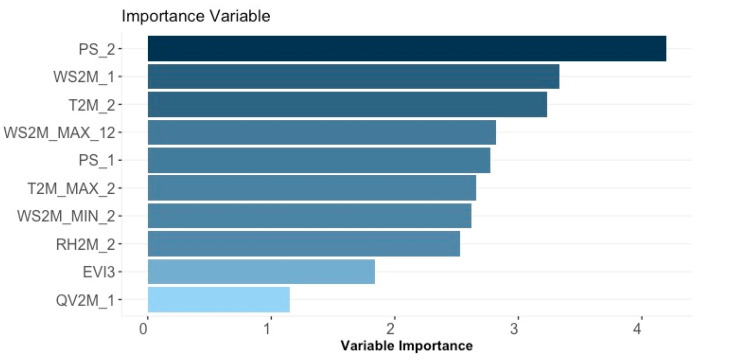
Feature importance based on Random Forest. The top 10 important features for total honey harvest prediction are reported. PS2: Average of surface pressure at the surface of the earth (kPa) in February; WS2M_1: Mean wind speed (*m*/*s*) at 2 m in January; T2M_2: Mean temperature (°C) at 2 m in February; WS2M_MAX_12: Maximum wind speed (*m*/*s*) at 2 m in December; PS_1: Average of surface pressure at the surface of the earth (kPa)in January; T2M_MAX_2: Maximum temperature (°C) at 2 m in February (2); WS2M_MIN_2: Minimum wind speed (*m*/*s*) at 2 m in February (2); RH2M_2: Mean relatives humidity (%) at 2 m in February; EVI3: Enhanced Vegetation Index in March; QV2M_1: Mean specific humidity (g/kg) at 2 m in January (1).

Training a model with redundant or uninformative features can be ineffective and inefficient. Indeed, non-informative features cannot contribute to improving the performance of a predictive model. The Boruta algorithm identified irrelevant features, enabling unbiased and efficient model training, and these features were subsequently removed from the original dataset. The 18 remaining variables included in the training and testing sets were: QV2M_1, T2MDEW_1, RH2M_1, WS2M_MIN_2, RH2M_2, WS2M_MAX_12, WS2M_1, PS_1, T2M_2, T2M_MAX_2, EVI3, PS_2, WS2M_MAX_1, T2M_MAX_1, WS2M_MAX_2, T2M_MIN_1, T2M_1 and PRECTOTCORR_1. After employing Boruta for feature selection, we excluded non-informative or redundant variables, thus refining the final set of predictors used for model training. To ensure the consistency of performance metrics, we compared three model configurations (see Figure S1—Supplementary Material SC): (i) a full model using all available variables, (ii) a model using only the features retained by Boruta, and (iii) a reduced model using only the six most influential variables based on Shafigurepley importance scores (see Figure 2). Notably, the Boruta-selected subset offered comparable AUC while preserving interpretability, indicating that the exclusion of low-importance features did not compromise predictive performance. In fact, reducing the feature set mitigated the risk of overfitting and decreased computational overhead, reinforcing that the retained predictors were both statistically significant and practically relevant for accurately forecasting honey production.

### 3.3. Model Training and Evaluation on Training and Test Sets

Three algorithms (DT, RF, and XGBoost) were applied to the dataset, and their predictive performance on both training and testing sets was compared according to several metrics. The hyperparameters were optimized for the three ML algorithms (Table 3) using GridSearch, a method that systematically explores a predefined set of parameter combinations. This approach ensures the selection of the best configuration by evaluating performance through cross-validation. Each algorithm had different hyperparameters that were tuned to achieve optimal performance. Thus, for RF, the number of variables randomly sampled as candidates at each split (mtry) was set to 7, and the minimum number of data points in a node (min_n) was set to 22, ensuring robust splits and avoiding overfitting. Instead, the optimal DT model was found with a cost_complexity of 0.00000353 to control tree pruning, and min_n was 19. For XGBoost, the tuned model used a more complex set of hyperparameters, including trees (682), min_n (4), tree_depth (5), learn_rate (0.0424), loss_reduction (0.0000000464), and sample_size (0.958). The identification of optimized hyperparameters suggests that a model selection process was performed to find the best settings for each algorithm.

**Figure 3:**
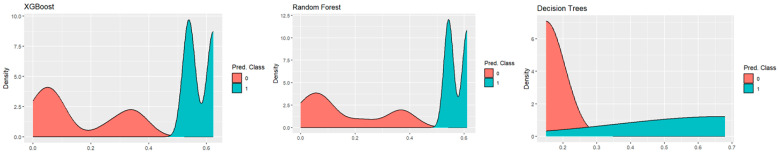
Prediction density plots for the test set showing the distribution of predicted probabilities for high and low honey production levels across the tree algorithms Decision Trees, Random Forest and Extreme Gradient Boosting.

Similar performances were observed for all three algorithms. Specifically, accuracy was 0.82 on the training set and 0.76 on the testing set. Specificity was greater than 0.92 in the training set and greater than 0.85 in the testing set. Sensitivity was only slightly lower in the testing set than in the training set (0.48 vs. 0.50, respectively). A similar result was found for precision: 0.85 in the training set vs. 0.83 in the testing set for all models. In addition, among the algorithms, the mean variation in AUC between the training and testing sets was close to 5%. Similarly, the AUC variation across the algorithms in the testing set was less than 20% (0.66 for DT vs. 0.82 for RF). Our results indicated that RF exhibited the best performance (AUC: 0.82), followed by XGBoost (AUC: 0.77), while DT showed a relatively lower performance (AUC: 0.66). Figure 3 presents the prediction density plots for the three algorithms (DT, RF, and XGBoost) based on the predicted probabilities from the test set. These probabilities represent the likelihood of each observation belonging to class 1 (high honey production) or class 0 (low honey production). The distributions of RF and XGBoost are visually comparable, showing a clear distinction between the probability densities of the two classes. In contrast, the DT model displays a greater overlap between these densities, which aligns with its lower AUC compared to RF and XGBoost. The predicted probabilities plotted are continuous values representing the likelihood of an observation belonging to class 1. Although the final classification is binary (high or low production), the continuous analysis of probabilities provides a more detailed understanding of the confidence level in the predictions. These results highlight the superior discriminative ability of RF and XGBoost, which more effectively separate high production levels from low ones, enhancing the reliability of predictions for the test data.

### 3.4. Feature Importance and Performance

Under the specified computational conditions (MacBook Pro [M1, 2020, 16 GB RAM]), we measured the execution time of three algorithms: Random Forest, XGBoost, and Decision Tree. RF exhibited a 28% reduction in execution time compared to XGBoost and a 55% reduction compared to DT. XGBoost also outperformed DT by 37% in terms of computational efficiency. Although DT had the fastest processing time, its predictive performance was lower. Detailed execution times are presented in Supplementary SB Table S1. Therefore, although XGBoost exhibited good predictive capacity, Random Forest (RF) demonstrated more robust performance—combining a higher AUC with faster processing—thus offering a more favorable balance between accuracy and computational cost. For this reason, we adopted RF as our primary tool for result interpretation and to guide the variable selection process. The feature importance analysis, conducted using the best-performing RF model, revealed the top 10 most influential factors that contribute to predicting THH (Figure 2). Among the 18 features retained after the feature selection process, the average temperature and pressure in February (T2M_2 and PS_2), as well as the average wind speed in January (WS2M_1), emerged as the most significant predictors.

In addition, we evaluated the impact of feature selection on model performance. Figure S1 (Supplementary Material SC) illustrates how the number of selected features influenced the predictive ability of the best-performing algorithm. Our results demonstrate that feature selection using the Boruta algorithm yielded the highest AUC, outperforming models trained with all features and a limited subset of top-ranked features. This finding reinforces the importance of an effective feature selection strategy for optimizing ML models and balancing model complexity and predictive accuracy.

## 4. Discussion

The application of machine learning algorithms has gained increasing attention in recent years due to their ability to capture intricate relationships between variables without requiring prior assumptions about their nature. Despite the relatively limited number of studies on honey bees, a range of inputs and outputs in beehive monitoring data models suggest that research has focused on the division between data collection and analysis, as well as determining the appropriate data to collect and how to obtain valuable insights. Most studies compare different approaches and algorithms to identify the best-suited ones for specific application cases. However, they often lack data from multiple beehives, apiaries, and geographical locations, leading to results that are specific to a particular condition and may differ when these limitations are addressed [59]. External validation is often recommended to guarantee the generalizability of ML models and reduce overfitting. In the present study, the original dataset was split into two subsets so that one subset was used to build and train the models, while the other was excluded from model building and used as an external testing set to evaluate the model’s performance on unseen data. Nevertheless, we are aware that future studies should evaluate the model’s performance on other data (e.g., bees experiencing different climatic conditions) to generalise our findings to other contexts or bee populations. In our study, we found that climatic conditions during the coldest months prior to harvest can indicate that changes in hive comfort may reduce production and colony survival levels. In particular, we found that atmospheric pressure, wind, temperature, humidity during the coldest months, and EVI at the beginning of the harvest season are good predictors of high or medium-low honey production. These findings align with recent studies that have highlighted the critical role of weather conditions during the winter months in forecasting agricultural yields, such as vegetative production [60,61,62]. The elevated temperatures and pressure levels in February may signal the onset of favorable growing conditions for nectar-producing plants, while the wind patterns in January could impact the foraging behavior and productivity of honey bees.

To our knowledge, this is the first study to predict honey harvest using climatic and environmental information prior to harvest, making it difficult to compare with previous research. These findings can be attributed to the fact that Lombardy, one of the coldest regions of Italy, has a climate that largely corresponds to Central European weather conditions. The region experiences cold and wet conditions from November to March, with the majority of the rainfall occurring from April to May. In the timeframe 2014–2019, the atmospheric pressure was greater from November to February than the rest of the year, and it affected nectar secretion and foraging activity. Additionally, the mean temperature trend showed monthly temperatures below 6 °C in winter, with variations ranging from 10 °C in 2017 to 15 °C during the same season for all years. In contrast, the relative humidity trend was higher in the winter months from 2014 to 2019. Temperature and humidity have a significant impact on thermoregulation [3,63,64], brood rearing [65,66], drone rearing [67], food storing and brood rearing activity [68], and matting flights [69,70], which have a direct relation with honey yield. In addition, low temperatures have been linked to a reduction in the number of adult workers [71], parasite infection in summer [72], flight reduction [73], and starvation [74]. Other studies have identified the effect of biotic and abiotic factors on bees’ foraging [75,76,77], reproduction [78], health status [79], genetics [80], behaviour [81] and yield [43,46,47] during the honey flow period. In particular, Campbell et al. [43] used regression trees to estimate honey harvest in South West Australia, utilizing weather and vegetation-related datasets from satellite sensors. The authors concluded that data from the month immediately preceding honey flow, out of the 115 input features collected both prior to and during the harvest period, is sufficient to make accurate predictions with minimal reduction in model performance. Additionally, Karadas et al. [48] also included anthropogenic features to predict honey production.

Therefore, monitoring winter weather patterns and leveraging advanced ML methods can help beekeepers more accurately anticipate critical hive conditions, such as changes in temperature, humidity, and atmospheric pressure. These insights enable the implementation of targeted strategies, such as adjusting feeding regimes, introducing supplementary heating, or adopting other climate-adaptive measures based on forecasted conditions. This study underscores the potential of ML in apiculture, serving as a foundational step toward future research aimed at developing practical tools for beekeepers. While the current findings demonstrate that ML can effectively predict honey production based on climatic and environmental factors, they also highlight the need for further refinement to enhance model accuracy and applicability. Incorporating additional variables, such as floral diversity, pest prevalence, and management practices, could lead to more robust predictions and facilitate the development of user-friendly decision-support systems. Similar approaches, as evidenced by Campbell et al. [43], Switanek et al. [13], and Karadas et al. [48], demonstrate how predictive analytics can bridge the gap between theoretical models and practical applications, paving the way for more precise and adaptive beekeeping strategies.

### Scalability and Generalizability

Our study provides new insights into how winter climate and environment affect honey production, but its regional specificity requires further exploration. To enhance generalizability and predictive capacity, future research should apply our approach to a database encompassing diverse geographic regions and incorporate additional factors, like as floral diversity (species richness, bloom timing, etc.), the prevalence of pests and diseases (e.g., Varroa mites, specific viral or bacterial infections), and beekeeping management practices (hive management techniques, supplemental feeding, migratory patterns). This broader approach will enable the development of more robust and comprehensive predictive models, ultimately leading to improved honey production management in various environmental contexts, including those affected by climate change.

## 5. Conclusions

Honeybee populations are increasingly threatened by climate change, experiencing escalating stressors that have reduced the productivity of managed colonies. These stressors affect bee survival by influencing the availability and quality of floral resources, directly impacting bee behavior and physiology, and affecting colony development and honey production. Furthermore, shifting climate conditions alter honeybee distribution, creating new competitive interactions among species, ecotypes, parasites, and pathogens. Precision beekeeping offers a vital strategy to support honeybees in this changing environment. By leveraging ML and innovative technologies, precision beekeeping empowers data-driven decision-making. Beekeepers can use pre-harvest weather parameters, such as winter temperature, humidity, atmospheric pressure, and wind speed, to inform management strategies for mitigating adverse conditions. Incorporating insights into optimal hive relocation sites, a common practice in migratory beekeeping can further enhance these decisions. Moreover, integrating additional factors into predictive models, such as flower species, management practices, pesticide/herbicide exposure, and the beekeepers’ technological capabilities, can improve prediction accuracy. Machine learning algorithms, particularly Random Forest, show promise for future research on honey harvest prediction. These data-driven approaches enable more resilient management strategies, allowing beekeepers to minimize losses and maintain productivity despite the challenges of a changing climate. Continued research, integrating these insights with landscape genomics, will further refine our understanding of bee adaptation and inform conservation efforts, ensuring the long-term health of bee populations, ecosystem services, and honey production.

## Figures and Tables

**Table 1:** Features related to monthly climatic conditions of winter prior to harvest and environmental information of harvesting season.

Time Period	Features	Abbreviation
Winter prior to harvest	Mean temperature (°C) at 2 m in December (12), January (1) and February (2)	T2M
	Maximum temperature (°C) at 2 m in December (12), January (1) and February (2)	T2M_MAX
	Minimum temperature (°C) at 2 m in December (12), January (1) and February (2)	T2M_MIN
	Mean drew/frost point temperature (°C) at 2 m in December (12), January (1) and February (2)	T2MDEW
	Mean specific humidity (g/kg) at 2 m in December (12), January (1) and February (2)	QV2M
	Mean relatives humidity (%) at 2 m in December (12), January (1) and February (2)	RH2M
	Bias corrected average of total precipitation at the surface of the earth in water mass (mm/day) in December (12), January (1) and February (2)	PRECTOTCORR
	Average of surface pressure at the surface of the earth (kPa) in December (12), January (1) and February (2)	PS
	Mean wind speed (*m*/*s*) at 2 m in December (12), January (1) and February (2)	WS2M
	Maximum wind speed (*m*/*s*) at 2 m in December (12), January (1) and February (2)	WS2M_MAX
	Minimum wind speed (*m*/*s*) at 2 m in December (12), January (1) and February (2)	WS2M_MIN
Harvesting season	Enhanced Vegetation Index in March (3), April (4), May (5), June (6), and July (7)	EVI

**Table 2:** Descriptive statistics of total honey harvest (THH), climatic features of winter prior to harvest, and enhanced vegetation index (EVI) of harvesting season.

Time Period	Variable	Mean (SD)	Percentiles				
			p0	p25	p50	p75	p100
Winter prior to harvest to February)	THH	23.40 (11.40)	5.2	14.6	21.2	31.2	63.2
	T2M_12	4.48 (1.020)	2.65	4.03	4.76	5.11	5.9
	T2M_MAX_12	14.00 (1.66)	10.5	14.3	14.8	14.8	15.2
	T2M_MIN_12	−2.59 (1.90)	−5.25	−3.98	−2.31	−0.65	−0.45
	T2MDEW_12	0.70 (1.59)	−1.74	−0.1	0.32	2.39	2.82
	QV2M_12	4.16 (0.49)	3.48	3.91	3.97	4.76	4.76
	RH2M_12	77.70 (3.96)	74.2	74.2	76.1	81.6	83.4
	PRECTOTCORR_12	1.29 (1.20)	0.13	0.13	0.68	2.53	2.92
	PS_12	99.8 (0.52)	99.2	99.3	99.6	100	101
	WS2M_12	1.05 (0.15)	0.76	0.99	1.08	1.12	1.26
	WS2M_MAX_12	4.03 (0.49)	3.23	3.63	4.15	4.36	4.69
	WS2M_MIN_12	0.06 (0.02)	0.04	0.05	0.05	0.06	0.11
	T2M_1	2.95 (1.53)	0.86	0.86	3.21	3.65	5.37
	T2M_MAX_1	12.4 (2.36)	8.77	8.77	12.7	14.4	15.1
	T2M_MIN_1	−3.85 (1.93)	−6.53	−6.53	−4.03	−2.65	−0.88
	T2M_DEW_1	−1.48 (2.19)	−4.39	−4.39	−0.82	−0.16	1.74
	QV2M_1	3.59 (0.60)	2.81	2.81	3.78	3.91	4.52
	RH2M_1	74.10 (4.03)	69.2	69.2	76.5	77.2	79.1
	PRECTOTCORR_1	1.02 (0.66)	0.23	0.23	1	1.63	1.86
	PS_1	99.20 (0.28)	98.8	99.1	99.2	99.6	99.6
	WS2M_1	1.29 (0.09)	1.15	1.21	1.3	1.35	1.4
	WS2M_MAX_1	5.67 (1.25)	4.29	4.29	5.55	6.48	7.66
	WS2M_MIN_1	0.052 (0.02)	0.02	0.03	0.05	0.07	0.09
	T2M_2	4.93 (1.44)	2.8	3.57	5.92	6.14	6.31
	T2M_MAX_2	15.1 (3.31)	11.5	11.7	15	19.2	19.4
	T2M_MIN_2	−2.77 (2.49)	−5.52	−5.47	−2.17	0.65	0.65
	T2MDEW_2	0.77 (1.47)	−1.2	−0.23	0.28	2.6	2.6
	QV2M_2	4.2 (0.42)	3.66	3.91	4.03	4.7	4.7
	RH2M_2	76.40 (4.52)	67.8	76.6	77.4	80.9	80.9
	PRECTOTCORR_2	2.95 (1.54)	1.09	1.78	2.42	4.41	5.46
	PS_2	99.10 (0.38)	98.8	98.8	98.9	99.4	99.8
	WS2M_2	1.33 (0.15)	1.2	1.2	1.3	1.38	1.66
	WS2M_MAX_2	5.28 (1.02)	4.21	4.21	6.11	6.12	6.6
	WS2M_MIN_2	0.057 (0.03)	0.02	0.03	0.04	0.09	0.09
Harvest Season	EVI3	0.42 (0.09)	0.22	0.33	0.46	0.49	0.51
	EVI4	0.52 (0.09)	0.31	0.5	0.54	0.59	0.62
	EVI5	0.49 (0.11)	0.24	0.43	0.51	0.58	0.64
	EVI6	0.54 (0.07)	0.42	0.49	0.52	0.64	0.64
	EVI7	0.55 (0.05)	0.42	0.53	0.53	0.58	0.66

**Table 3:** Optimized hyperparameters for Random Forest, Decision Trees, and XGBoost for Honey harvest prediction.

Algorithm	Hyperparameters *	Values
DT	cost_complexity	3.53 × 10^−6^
	min_n	19
RF	Mtry	7
	min_n	22
XGBoost	Trees	682
	min_n	4
	tree_depth	5
	learn_rate	0.0424
	loss_reduction	4.64 × 10^−7^
	sample_size	0.958

* cost_complexity: refers to cost complexity pruning. Pruning is a technique that reduces the size of decision trees by removing branches that have little importance; min_n: minimum number of samples needed to make a split at a node; mtry: number of features considered for splitting a node in each decision tree; tree_depth: The maximum depth allowed for each decision tree; learn_rate: The learning rate of the model; loss_reduction: the minimum reduction in the loss metric needed to make a split at a node; sample_size: the percentage of samples that will be used to train each tree.

## Data Availability

The phenotypes are part of a reference population used for selection by a commercial breeder and have commercial value. Therefore, restrictions apply to the availability of these data, which are not publicly available. The authors can be contacted for specific requests.

## Supplementary Materials

The following supporting information can be downloaded at: https://www.mdpi.com/article/10.3390/insects16030278/s1. Supplementary File SA: Pseudo Code; Supplementary SB table S1: Computational Resources and Processing Time; Supplementary Material SC Figure S1: Trade-off between Area Under the ROC Curve (AUC) vs. Number of Features for Random Forest.
