# Supplementary material for: Combining Environmental Variables and Machine Learning Methods to Determine the Most Significant Factors Influencing Honey Production
Source: Insects. 2025 Mar 6;16(3):278. doi: 10.3390/insects16030278 (PMC11943014; doi:10.3390/insects16030278)
Supplement: Supplementary file 1 [file insects-16-00278-s001.zip › insects-3381750-supplementary.pdf]

Supplementary material for "Combining environmental variables and machine learning methods to determine the most significant factors influencing honey production"

A. Pseudo Code

START
1. Importing Packages
IMPORT necessary libraries (tidymodels, tidyverse, caret, Boruta….)
2. Data Loading and Preprocessing
LOAD dataset
TRANSFORM target variable (THH) into a binary class (0,1)
3. Feature Selection with Boruta
SPLIT data into training (75%) and testing (25%) sets (stratified by THH)
DEFINE preprocessing: z-score
RUN Boruta algorithm on the training set
IDENTIFY important features and discard irrelevant ones
4. Model Definition
CONFIGURE models:
- Decision Tree
- Random Forest
- XGBoost

DEFINE hyperparameters to be tuned (e.g., number of trees, tree depth, learning rate)
5. Hyperparameter Tuning via Cross-Validation
DEFINE cross-validation (5-fold and 10 repetitions, stratified by THH)
FOR EACH model:
- EXECUTE GridSearch to find optimal hyperparameters
- OPTIMIZE metrics (AUC, accuracy, specificity, sensitivity and Precision)

6. Final Training and Model Evaluation
SELECT best hyperparameters for each model
TRAIN final model using optimized data
EVALUATE performance on the test set
7. Feature Importance Analysis
GENERATE variable importance plot (VIP)
END

B. Computational Resources and Processing Time

Table S1: Computational cost (in seconds) for Random Forest (RF), Extreme Gradient Boosting (XGBoost) and Decision Trees (DT).

Algorithm	Computational Cost (s)
Decision Trees	12.2921
Random Forest	19.3123
Extreme Gradient Boosting - XGBoost	26.8128

C. Trade-off between Area Under the ROC Curve (AUC) vs. Number of Features for Random Forest

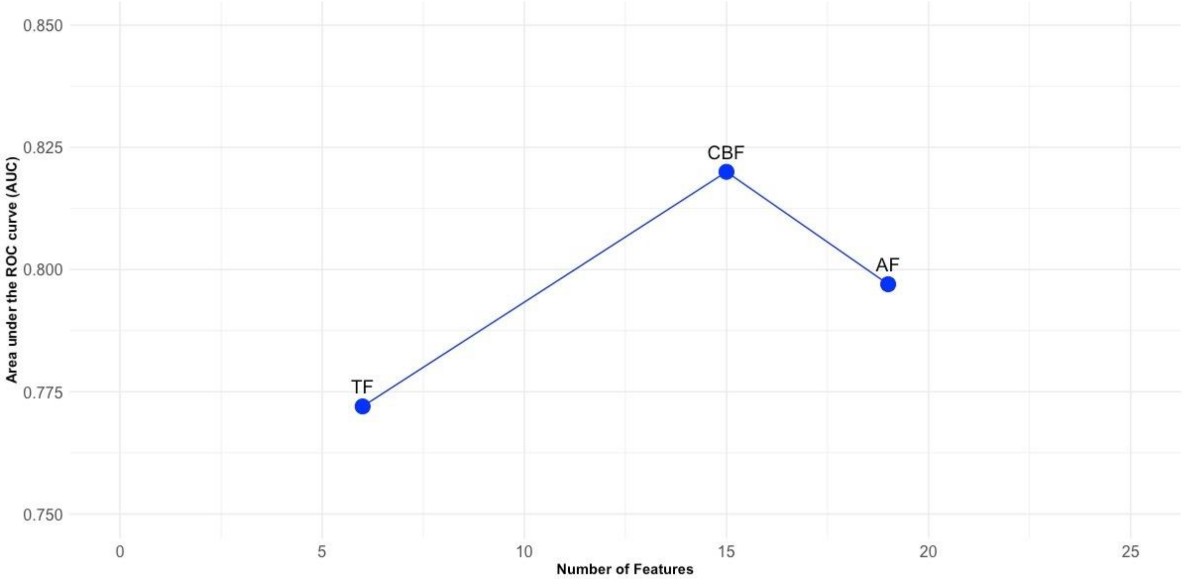

TF: Top features selected by the Boruta algorithm; CBF: Confirmed Boruta Features; AF: The original features (prior to feature selection).

Figure S1: Trade-off between Area under the ROC curve (AUC) vs. Number of Features for Random Forest.
